# Hrs Promotes Ubiquitination and Mediates Endosomal Trafficking of Smoothened in *Drosophila* Hedgehog Signaling

**DOI:** 10.1371/journal.pone.0079021

**Published:** 2013-11-11

**Authors:** Junkai Fan, Kai Jiang, Yajuan Liu, Jianhang Jia

**Affiliations:** Markey Cancer Center, Department of Molecular and Cellular Biochemistry, The University of Kentucky College of Medicine, Lexington, Kentucky, United States of America; Indiana University School of Medicine, United States of America

## Abstract

In Hedgehog (Hh) signaling, the seven-transmembrane protein Smoothened (Smo) acts as a signal transducer that is regulated by phosphorylation, ubiquitination, and cell surface accumulation. However, it is not clear how Smo cell surface accumulation and intracellular trafficking are regulated. Here, we demonstrate that inactivation of Hrs by deletion or RNAi accumulates Smo in the late endosome that is marked by late endosome markers. Inactivation of Hrs enhances the wing defects caused by dominant-negative Smo. We show that Hrs promotes Smo ubiquitination, deleting the ubiquitin-interacting-motif (UIM) in Hrs abolishes the ability of Hrs to regulate Smo ubiquitination. However, the UIM domain neither recognizes the ubiquitinated Smo nor directly interacts with Smo. Hrs lacking UIM domain still downregulates Smo activity even though to a less extent. We have characterized that the N-terminus of Hrs directly interacts with the PKA/CK1 phosphorylation clusters to prevent Smo phosphorylation and activation, indicating an ubiquitin-independent regulation of Smo by Hrs. Finally, we found that knockdown of Tsg101 accumulates Smo that is co-localized with Hrs and other late endosome markers. Taken together, our data indicate that Hrs mediates Smo trafficking in the late endosome by not only promoting Smo ubiquitination but also blocking Smo phosphorylation.

## Introduction

The Hedgehog (Hh) morphogen controls such development processes as cell proliferation, embryonic patterning, and cell growth [1]–[3]. Dysregulation of Hh signaling has been implicated in many human disorders, including several cancer types [4]–[6]. Smoothened (Smo), an atypical G protein-coupled receptor (GPCR), is essential in both insects and mammals for transduction of the Hh signal [2], [3], [7]. Abnormal Smo activation results in basal cell carcinoma (BCC) and medulloblastoma, so it remains an attractive therapeutic target.

In *Drosophila*, the Hh signal is transduced through a reception system at the plasma membrane, which includes the receptor complexes Ptc-Ihog and the signal transducer Smo [7]–[9]. Binding of Hh to Ptc-Ihog relieves the inhibition of Smo by Ptc, allowing Smo to ultimately activate the Cubitus interuptus (Ci)/Gli family of Zn-finger transcription factors and thereby induce the expression of Hh target genes, such as *decapentaplegic* (*dpp*), *ptc*, and *en* [7], [10]. Intracellular Smo is unstable and very low levels of Smo has been observed in the absence of Hh. Upon different levels of Hh stimulation, Smo is differentially phosphorylated [11], [12] and phosphorylation increases the level of cell surface Smo [2], [7], although the mechanism is not completely understood. Using an antibody uptake assay, we found that Hh treatment inhibited Smo endocytosis and reduced the ratio of early endosome-localized Smo [13]. In addition, ubiquitination promotes endocytosis of Smo, whereas deubiquitination prevents the process [13], [14]. A transmission electron microscopic study of *Drosophila* imaginal discs indicated that Smo is directed primarily to the lysosomes of A compartment cells, but is enriched at the plasma membrane of P compartment cells [15]. It is clearly important to understand how Hh regulates Smo trafficking and how ubiquitination promotes Smo endocytosis. Among the proteins that regulate receptor intracellular trafficking, the components from the Endosomal Sorting Complex Required for Transport (ESCRT) are critical. In *Drosophila*, the endosomal sorting of Smo is likely regulated by the *Drosophila* homolog of HGF-regulated tyrosine kinase substrate (Hrs), as Smo accumulation has been observed in cells mutating *hrs* [14], [16]. In mammals, Hh signal transduction depends on the primary cilium, and ciliary accumulation is required for Smo activation [17]–[19], thus the cilium represents a signaling center for Hh pathway in mammals [20]. Similarly, phosphorylation by multiple kinases promotes the ciliary localization of mammalian Smo [21]. However, the mechanisms by which the ciliary localization of vertebrate Smo is controlled remain unclear.

In this study, we provide genetic and biochemical evidence that the ubiquitination and intracellular trafficking of Smo are regulated by Hrs through direct interaction that inhibits the phosphorylation of Smo at the carboxyl-terminal domain. We further provide evidence that both Hrs and tumor susceptibility gene 101 (Tsg101) mediate Smo trafficking in the late endosomes, likely downstream of Shibire (Shi, the *Drosophila* homolog of the dynamin GTPase).

## Results

### Hrs Regulates Smo Activity through Mediating Smo Trafficking in the Late Endosome

**Figure 1:**
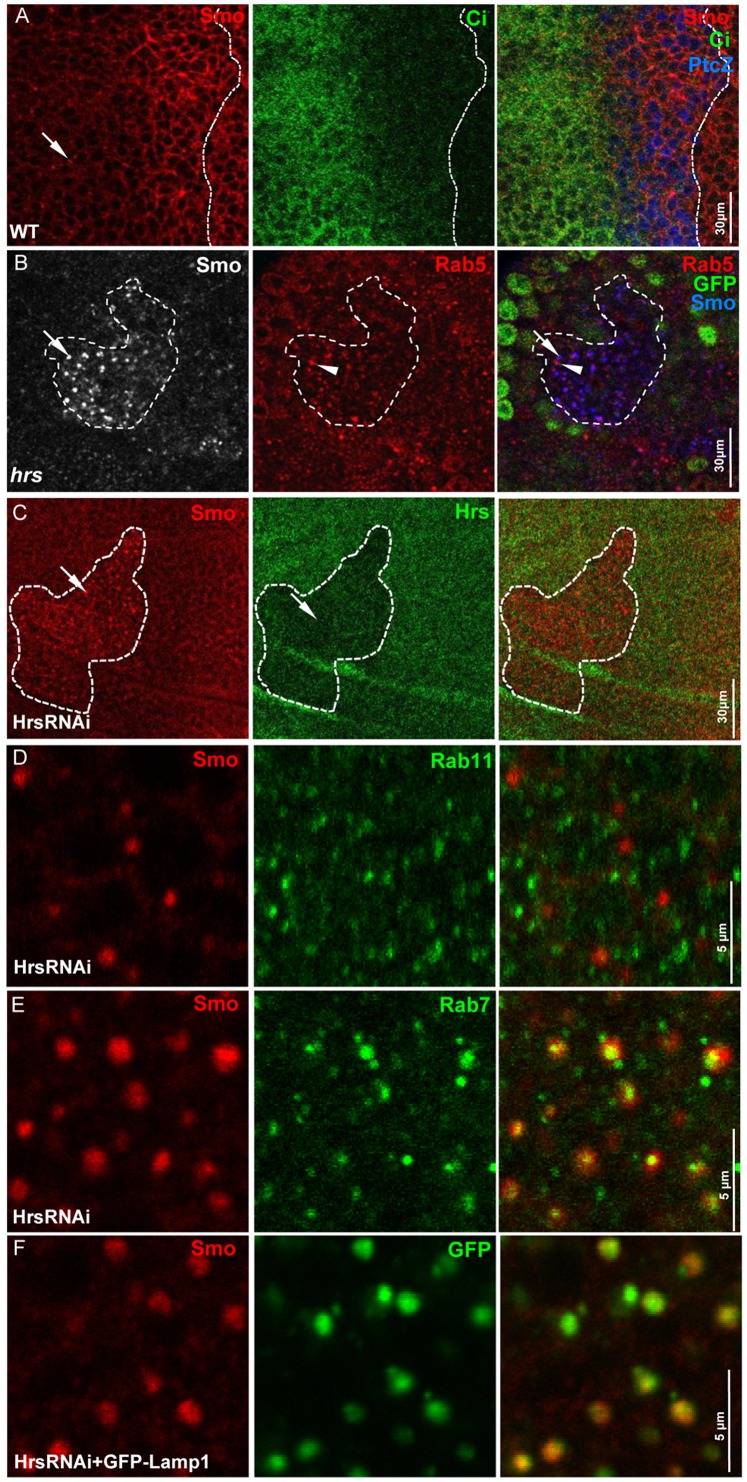
Inactivation of Hrs accumulates Smo. (A) A WT wing disc stained for Smo, Ci, and *ptc*-lacZ. Arrow indicates Smo puncta in A compartment cells where there is no Hh. White dashed line indicates the A/P boundary that is defined by Ci staining. (B) An *hrs* mutant clone marked by the lack of GFP expression was stained with the anti-Smo and anti-Rab5 antibodies. Arrows indicates the accumulated Smo and arrowheads indicate the early endosome labeled by Rab5. White dashed line marks the clone. Antibody staining outside the clone serves as control. (C) A wing disc expressing HrsRNAi by *act>CD2>*Gal4 was stained with anti-Smo and anti-Hrs antibodies. The marked low levels of Hrs label the HrsRNAi expressing clone (arrow in green color panel). HrsRNAi causes Smo accumulation in puncta (arrow in red color panel). White dashed line marks the clone. Antibody staining outside the clone serves as control. (D) High magnification image from a wing disc expressing HrsRNAi by the dorsal compartment-specific *ap*-Gal4 and stained for Smo and Rab11. (E) High magnification image from a wing disc expressing HrsRNAi by *ap*-Gal4 and stained for Smo and Rab7. (F) High magnification image from a wing disc coexpressing HrsRNAi with GFP-Lamp1 by *ap*-Gal4 and stained for Smo and GFP. All wing imaginal discs shown in this study were oriented with anterior on the left and ventral on the top.

Hh induces stabilization and accumulation of Smo at the cell surface [22], [23]. In *Drosophila* wing imaginal disc, the posterior (P) compartment cells express and secrete Hh proteins that act upon neighboring anterior (A) compartment cells located adjacent to the A/P boundary to induce the expression of Hh target genes. P-compartment cells as well as A-compartment cells near the A/P boundary exhibit high levels of Smo cell surface accumulation (Fig. 1A). In A-compartment cells away from the A/P boundary, Smo levels are extremely low and the intracellular puncta of Smo suggest the trafficking of Smo inside the cell that leads to degradation of the protein (Fig. 1A). We recently showed that ubiquitination promotes Smo intracellular trafficking that is mediated by endosomes [13]. It was also reported that Smo accumulates in cells mutating *hrs* that encodes a protein involved in sorting ubiquitinated membrane proteins in the endosomes [14], [16], raising the possibility that Hrs may facilitate endosomal sorting of Smo. Smo accumulated as puncta in mutant clones lacking *hrs* [14] (Fig. 1B). Inactivation of Hrs by RNAi also accumulates Smo in puncta (Fig. 1C). The phenotype of HrsRNAi is unlikely due to an off-target effect because expression of different transgenic lines from Bloomington (#28026 and #28964) and VDRC (v20933) targeting different non-overlapping regions produced a similar phonotype that is consistent with the phenotype caused by *hrs* mutation. We thus used the HrsRNAi lines to examine the localization of Smo when Hrs is inactivated. We found that Smo puncta co-localized neither with the early endosome marker Rab5 (Fig. 1B), nor with the recycling endosome marker Rab11 (Fig. 1D). Instead, Smo puncta co-localized with the late endosome marker Rab7 (Fig. 1E). In addition, Smo puncta co-localized with the overexpressed GFP-Rab7 and GFP-Lamp1 (Fig. 1F, data not shown), which are often expressed in the late endosomes. These data suggest that Hrs facilitates Smo sorting into the late endosome.

**Figure 2:**
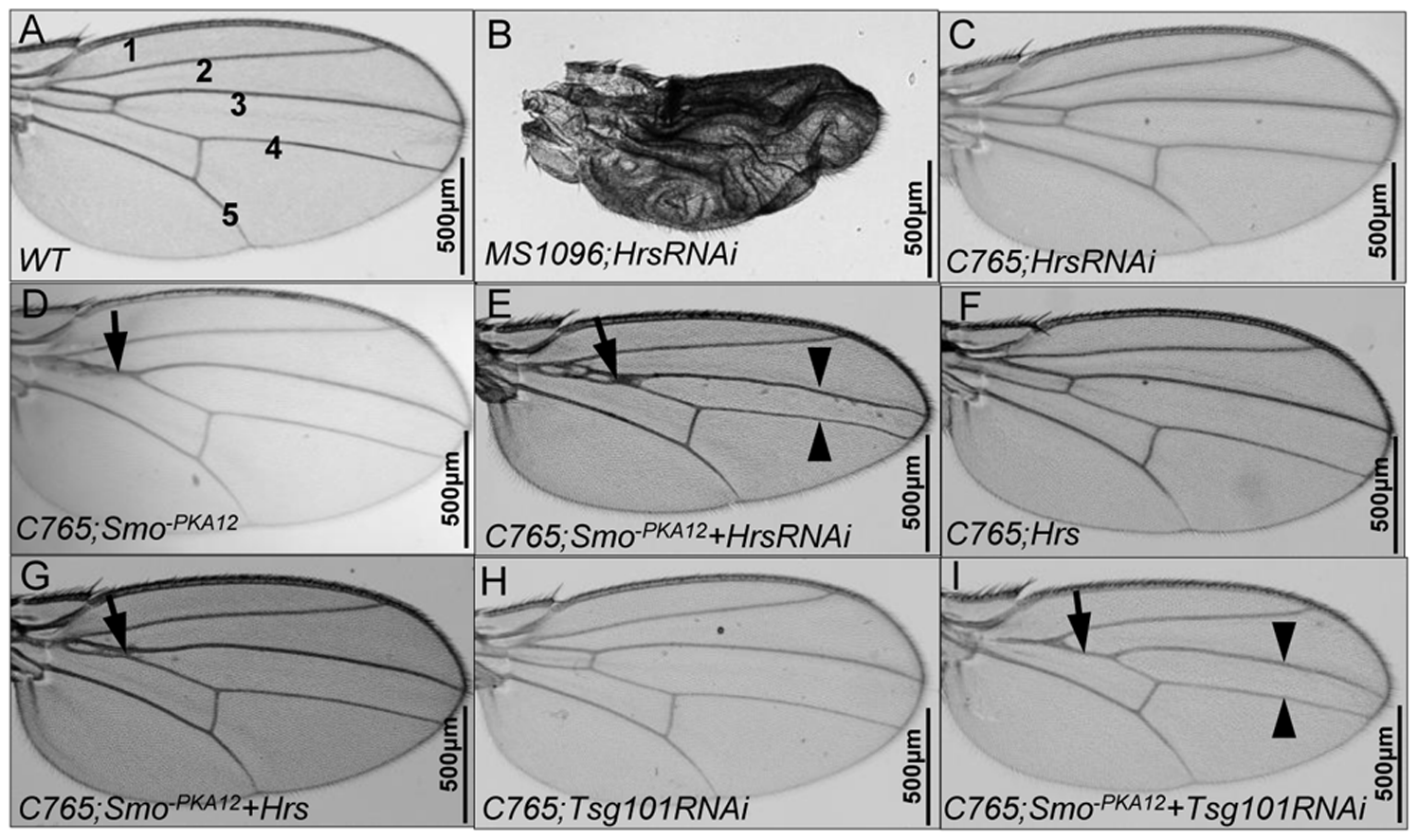
Hrs modifies the dominant-negative activity of Smo^−PKA12^. (A) A wild-type adult wing showing interveins 1-5. (B-C) Wings from flies expressing HrsRNAi by either the wing-specific *MS1096*-Gal4 or *765*-Gal4. (D-E) Wings from flies expressing either Smo^−PKA12^ alone or together with HrsRNAi by *C765*-Gal4. Arrows indicate the fusion of Vein 3 and 4 that is a partial loss of Hh phenotype. Arrowheads indicate the further fusion of Vein 3 and 4 that is caused by coexpression of HrsRNAi. (F-G) HA-Hrs was expressed either alone or together with Smo^−PKA12^ by *C765*-Gal4. Arrow indicates the weaker phenotype compared to D. (H–I) Tsg101RNAi was expressed either alone or in combination with Smo^−PKA12^ by *C765*-Gal4. Arrow and arrowheads indicate the enhanced fusion between Vein 3 and 4.

Knockdown of Hrs by RNAi using the wing-specific *MS1096*-Gal4 caused a severe phenotype in adult wings (Fig. 2B, compared to WT wing in Fig. 2A). To better explore the Hh wing phenotype and to examine whether Hrs plays a role in regulating Smo activity, we used a weaker Gal4 line, *C765*-Gal4. As we previously described, expressing the phospho-deficient Smo mutant, Smo^−PKA12^ (a weak dominant-negative form), by *C765*-Gal4 caused a reproducible wing phenotype with partial fusion between Vein 3 and 4 (arrow in Fig. 2D, compared to WT wing structure in Fig. 2A) [24]. This phenotype provided a sensitized genetic background for screening novel components involved in Hh signaling [24]. We reasoned that if Hrs regulates Smo activity in wing development, manipulating Hrs expression levels may dominantly modify this phenotype. Indeed, knockdown of Hrs by RNAi in Smo^−PKA12^ expressing wing caused further fusion and narrower Vein 3 and Vein 4 (Fig. 2E, compared to Fig. 2D), although HrsRNAi alone driven by the *C765*-Gal4 did not cause any phenotype in the wing (Fig. 2C). We further generated *UAS-HA-Hrs* transgene and assessed its ability to regulate Smo activity. We found that coexpressing Smo^−PKA12^ with HA-Hrs reduced the Smo^−PKA12^ phenotype (Fig. 2G), even though expressing HA-Hrs alone produced wild-type wings (Fig. 2F). These data suggest that changing Hrs levels in wing discs leads to changes in the dominant-negative activity of Smo^−PKA12^.

### Hrs Promotes the Ubiquitination of smo

**Figure 3:**
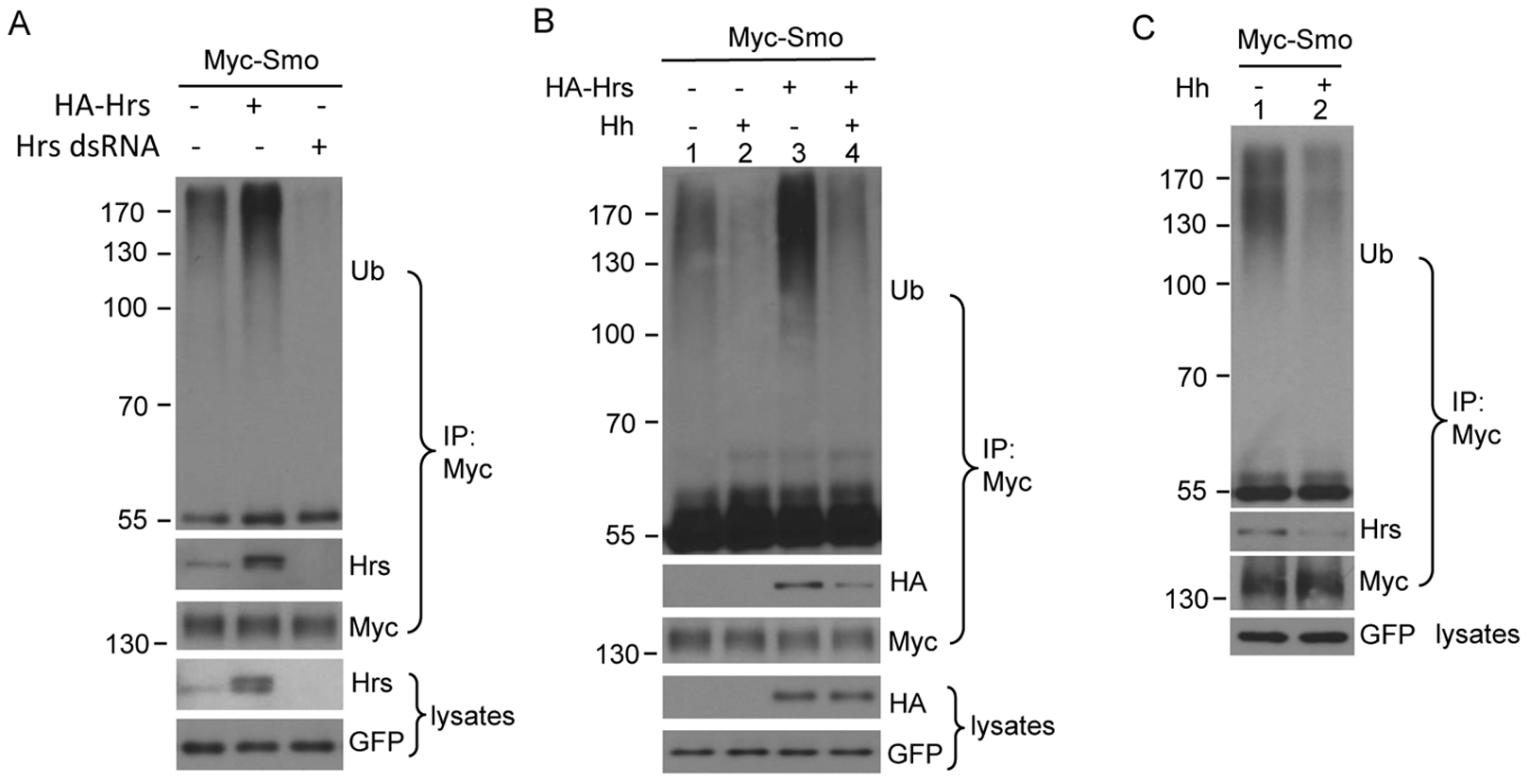
Hrs interacts with Smo and promotes Smo ubiquitination. (A) S2 cells were transfected with Myc-Smo alone or in combination with HA-Hrs or Hrs dsRNA. Cell extracts were subjected for immunoprecipitation with the anti-Myc antibody followed by western blots with anti-Ub, anti-HA, and anti-Myc to detect the ubiquitination of Smo, Smo-bound Hrs, and the levels of Myc-Smo, respectively. To normalize the levels of Smo, 50 mM MG132 and 15mM NH4Cl was used to block Smo degradation, and samples were normalized for loading. All figures in this study showing the levels of Myc-Smo were normalized by the same way. The efficiency of Hrs RNAi was determined by western blot with anti-Hrs antibody. GFP served as a transfection and loading control. (B) S2 cells were transfected with Myc-Smo alone or together with HA-Hrs, and treated with HhN-conditioned medium or control medium followed by immunoprecipitation and western blot with the indicated antibodies. Of note, the interaction between Smo and Hrs was down-regulated by Hh treatment. (C) S2 cells were transfected with Myc-Smo and treated with HhN-conditioned medium or control medium. Cell extracts were immunoprecipitated with the anti-Myc antibody and blotted with the indicated antibodies. Hh treatment reduced the interaction between Smo and endogenous Hrs.

We and others have shown that ubiquitination promotes Smo endocytic trafficking and that Hh prevent Smo endocytosis by inhibiting Smo ubiquitination [13], [14]. However, the machinery responsible for Smo ubiquitination is not known, although inactivation of the ubiquitin activating enzyme Uba1 downregulates Smo ubiquitination [14]. Since Hrs is a key endocytic regulator recognizing the ubiquitinated receptors and mediates receptor sorting onto multivesicular bodies [25], [26], we wondered whether Hrs regulates Smo by conserved mechanisms. Surprisingly, we found that Hrs regulates the levels of Smo ubiquitination even though Hrs is unlikely the ubiquitin-ligase for Smo. We examined Smo ubiquitination in S2 cells using the immunoprecipitation assay we have established [13]. The ubiquitination of Smo was readily detected by the anti-Ub antibody that recognized the immunoprecipitated endogenous Ub (Fig. 3A, top panel). Interestingly, the levels of Smo ubiquitination were increased by overexpression of Hrs, and decreased by RNAi of Hrs (Fig. 3A, top panel), indicating the involvement of Hrs in the process of Smo ubiquitination.

Hh treatment resulted in a reduction of Smo ubiquitination (Fig. 3B, top panel), which was consistent to our previous observations [13]. To determine whether Hrs has a role in the Hh-mediated reduction of Smo ubiquitination, we examined Smo ubiquitination when Hrs was coexpressed in S2 cells. We found that Hh-treatment consistently downregulated the levels of Smo ubiquitination in both Hrs overexpressing and non-overexpressing cells (Fig. 3B, top panel), suggesting that the Hrs-mediated Smo ubiquitination is downregulated by Hh.

In the above experiment examining Smo ubiquitination regulated by Hrs, we immediately detected the Smo-bound Hrs in the immunoprecipitation assay (Fig. 3A, 2^nd^ panel; Fig. 3B, 2^nd^ panel), suggesting that Smo and Hrs exist in the same protein complex. Furthermore, we found that the physical interaction between Smo and Hrs was inhibited by Hh-treatment (Fig. 3B, 2^nd^ panel), indicating that the downregulation of Smo ubiquitination correlates with the disassociation of Hrs. Consistently, Smo pulled down much less endogenous Hrs in the presence of Hh treatment (Fig. 3C, 2^nd^ panel). It is possible that Hh downregulates Smo ubiquitination by inhibiting the physical interaction between Smo and Hrs.

### The Function of Hrs Domains in Regulating Smo Ubiquitination

**Figure 4:**
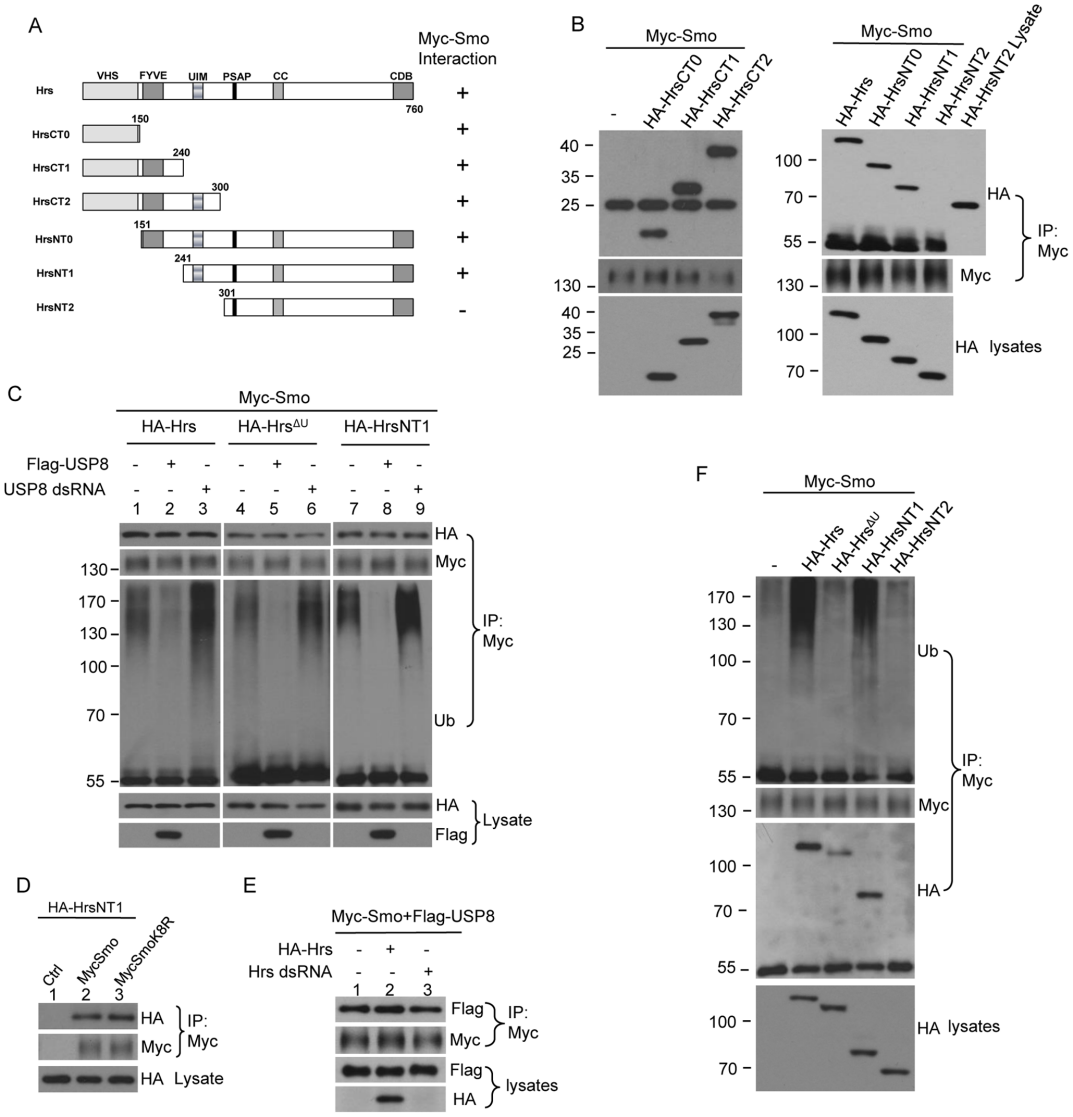
Hrs UIM and N-terminal domains physically interact with Smo, but only the UIM domain is required for promoting Smo ubiquitination. (A) A schematic drawing of Hrs truncations and their ability to interact with Myc-Smo in the immunoprecipitation assay described in this Figure. (B) S2 cells were transfected with Myc-Smo and the indicated Hrs constructs followed by immunoprecipitation and western blot with the indicated antibodies. Cell lysates were also subjected to western blot to examine the expression of Hrs full-length and Hrs truncations. The bands at 25kD in the top left panel and the bands at 55kD in the top right panel indicate the IgG that served as loading control. In the last lane of top right panel, the lysate of HA-HrsNT2 was loaded in order to show the absence of the same band in the adjacent lane. (C) S2 cells were transfected with Myc-Smo and HA-Hrs constructs in combination with Flag-USP8 or USP8 RNAi to examine whether changing ubiquitination levels of Smo by USP8 could alter the interaction between Smo and Hrs. Cell extracts were immunoprecipitated by the anti-Myc antibody followed by western blot with either the anti-HA or the anti-Myc antibody to examine the amount of Smo-bound Hrs and the levels of Smo. Western blot of the cell lysates was to examine the expression of Hrs or USP8. (D) S2 cells were co-transfected with HA-HrsNT1 that contains the UIM domain and Myc-Smo or Myc-SmoK8R that bears K>R mutation in the domain binding Hrs. The immunoprecipitation assay was performed with the anti-Myc antibody and the Smo-bound Hrs was examined by the anti-HA antibody. K>R mutation did not change the interaction between Smo and HrsNT1. (E) S2 cells were transfected with the indicated constructs or Hrs dsRNA. Cell extracts were subjected to immunoprecipitation with the anti-Myc antibody followed by western blot with the anti-Myc or anti-Flag antibodies. Cell lysates were subjected to direct western blot with the anti-Flag and anti-HA antibodies to examine the protein expressed. (F) S2 cells were transfected with Myc-Smo and Hrs variants to examine the ability of different forms of Hrs in regulating Smo ubiquitination.

To further assess the function of Hrs in regulating Smo ubiquitination, we generated hemagglutinin (HA)-tagged Hrs truncations (Fig. 4A) and examined their interaction with Smo as well as their effects on Smo ubiquitination in cultured S2 cells. We carried out a series of immunoprecipitation experiments to map the Hrs domain that is responsible for the interaction with Smo. Collectively, the data showed that the region aa 1-300 consisting of the VHS, FYVE, and ubiquitin-interacting-motif (UIM) domains physically interacted with Smo (Fig. 4A-B), and that Hrs lacking the VHS, FYVE, and UIM domains (HrsNT2) did not interact with Smo (Fig. 4B, top panel).

Hrs normally interacts with ubiquitinated cargoes through its UIM domain and plays an essential role in endosomal sorting. We wondered whether it would be possible for Hrs to recognize the ubiquitinated Smo. Surprisingly, we found that the interaction between Smo and Hrs was not changed (Fig. 4C, top panel) when manipulating the levels of Smo ubiquitination by either overexpression or RNAi of USP8 (Fig. 4C, 3^rd^ panel), a deubiquitinase that has been characterized to downregulate Smo ubiquitination [13], [14]. In addition, the interactions between Smo and Hrs lacking the UIM domain (Hrs^ΔU^) or Hrs lacking the N-terminal VHS and FYVE domains but containing the UIM domain (HrsNT1) were not regulated by USP8 either (Fig. 4C, top panels). These data suggest that changing the levels of Smo ubiquitination does not change the interaction between Smo and Hrs. Of note, HrsNT2 did not interact but HrsNT1 interacted with Smo, suggesting that the UIM domain physically interacts with Smo, either directly or indirectly. We further carried out series immunoprecipitation experiment and mapped the domain in Smo that interacts with Hrs (see below). In this domain of Smo, mutation of eight lysine residues that are known for Smo ubiquitination [14] did not change the binding property (Fig. 4D, top panel). SmoK13R that contains 13 lysine residues supposed to be ubiquitinated [14] did not change Smo-Hrs interaction either (data not shown). We also found that the interaction between Smo and USP8 was not changed by either overexpression or RNAi of Hrs (Fig. 4E), suggesting that Hrs regulates Smo ubiquitination not by preventing the binding of the deubiquitinase to Smo.

**Figure 5:**
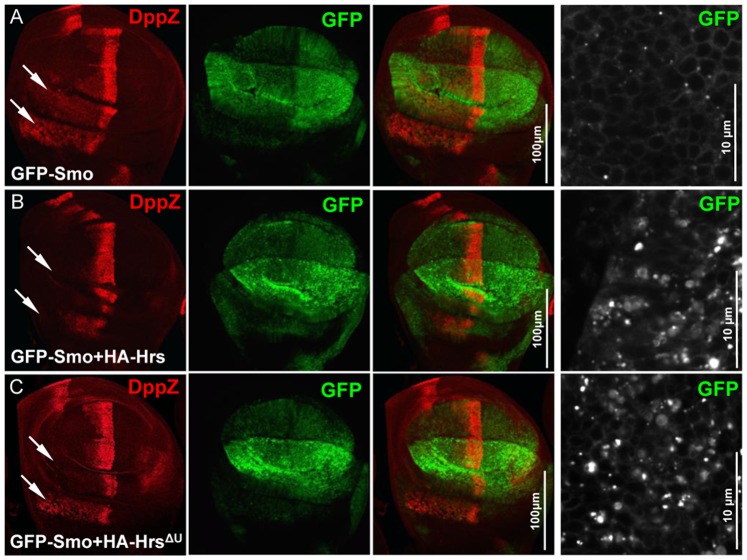
Hrs downregulates Smo activity in vivo. (A-C) Wing discs expressing GFP-Smo alone or in combination with HA-Hrs or HA-Hrs^ΔU^ were stained for *dpp*-lacZ expression. High magnification views are shown in the right column. The expression of both Hrs and Hrs^ΔU^ led to GFP-Smo puncta in wing discs.

We next examined Hrs variants for their ability to regulate Smo ubiquitination. The overexpression of full-length Hrs consistently elevated the levels of Smo ubiquitination (Fig. 4F, top panel). However, the expression of Hrs^ΔU^ lacking the UIM domain had much less effect on Smo ubiquitination (Fig. 4F, top panel). Consistently, the expression of HrsNT1 elevated Smo ubiquitination but the expression of HrsNT2 had no effect on Smo ubiquitination (Fig. 4F, top panels). We further generated Hrs^ΔU^ transgenic fly line to assess its in vivo activity in regulating Smo. The expression of GFP-Smo induced ectopic *dpp*-lacZ expression in cells away from the A/P boundary (Fig. 5A), which was diminished by the co-expression of Hrs (Fig. 5B). The activity of Hrs suppressing GFP-Smo activity was severely compromised by deletion of the UIM domain in Hrs (Fig. 5C). Since both Hrs and Hrs^ΔU^ were expressed at the VK5 attP locus, the effects were not due to the expression levels. These data suggest that the UIM domain of Hrs inhibits Smo activity through promoting Smo ubiquitination. UIM domain may binds ubiquitin in the other protein, such as a Smo E3 ligase that is necessary for Smo ubiquitination. Taken together, UIM is a highly conserved sequence that can bind ubiquitinated cargos and is found in a number of proteins involved in endocytosis and protein trafficking, and our results further indicate that it also functions to promote the ubiquitination of a target protein.

The finding that Hrs^ΔU^ was still able to downregulate GFP-Smo activity, even though to a lesser extent compared to full-length Hrs (Fig. 5C, compared to Fig. 5A), suggest that other domain(s) in Hrs regulates Smo activity. In support of this model, both Hrs and Hrs^ΔU^ caused GFP-Smo to form puncta in wing discs (Fig. 5, right column). Since Hrs^ΔU^ did not have potent activity in promoting Smo ubiquitination (Fig. 4F) but can still downregulate Smo activity in vivo, our findings suggest a novel ubiquitin-independent mechanism involved in Hrs-mediated Smo regulation. It is possible that the N-terminal VHS and FYVE domains of Hrs inhibit Smo activity because these domains physically interact with Smo (Fig. 4A–B).

### Hrs Prevents Smo Phosphorylation through Direct Interaction with the Phosphorylation Clusters of Smo

**Figure 6:**
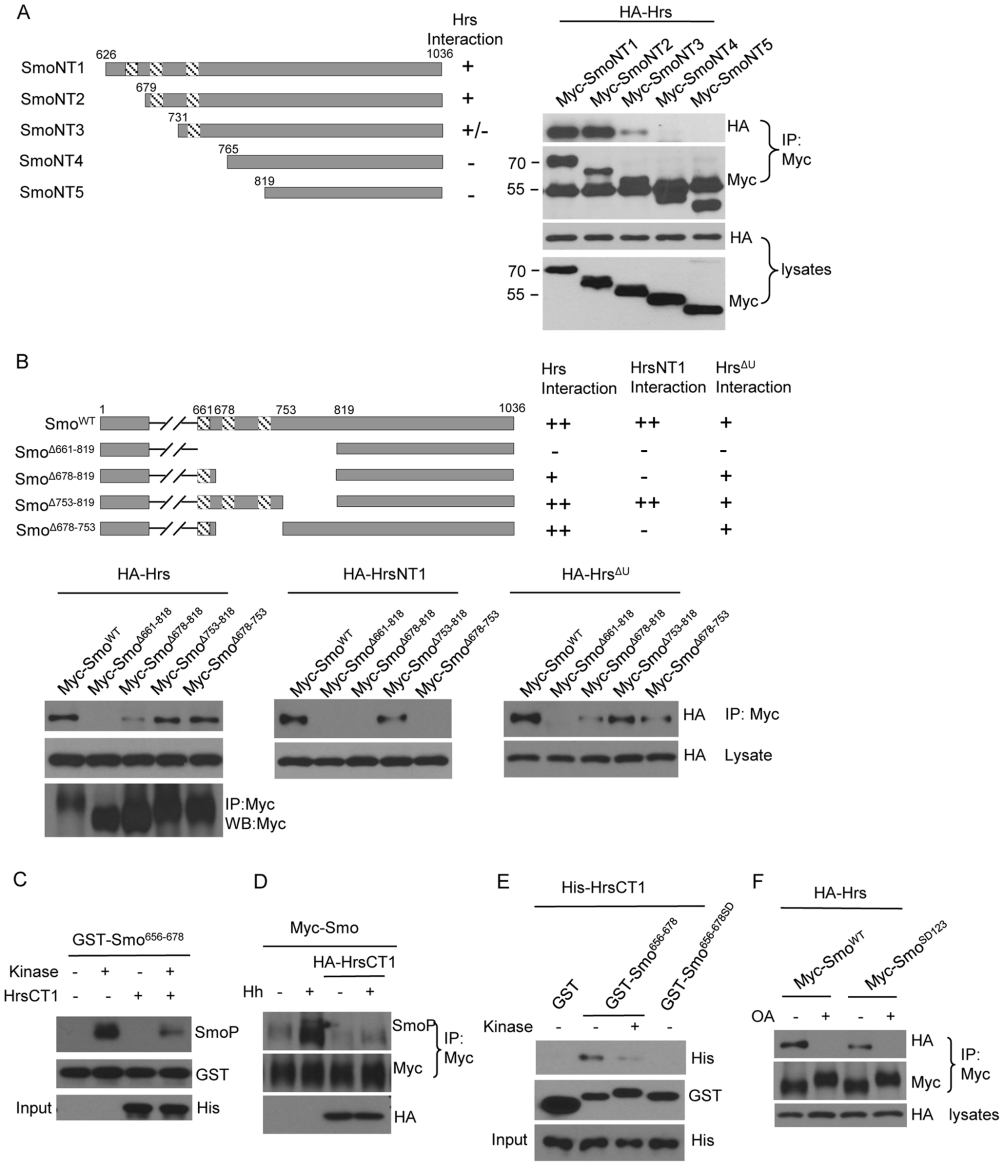
Direct interaction of Hrs N-terminal domains with Smo phosphorylation clusters blocks Smo phosphorylation. (A) A schematic drawing of Smo C-tail truncations and their interaction with Hrs. In the right panel, HA-Hrs was transfected in S2 cells with a series of Smo truncations. Cell extracts were immunoprecipitated with the anti-Myc antibody and subjected to a western blot with the anti-HA or anti-Myc. (B) A series of Smo internal deletion constructs was transfected in S2 cells with HA-Hrs, HrsNT1 or Hrs^ΔU^. Cell extracts were immunoprecipitated with the anti-Myc antibody and subjected to a western blot with anti-HA or anti-Myc. Cell lysates were also subjected to western blot to examine the expression of Hrs constructs. Myc-Smo levels are not shown in the bottom right panels. (C) An in vitro kinase assay using the purified GST-Smo^656−678^ with or without the kinase set (PKA and CK1). GST-Smo^656−678^ phosphorylation was detected by western blot with the anti-SmoP antibody. The input of bacterially expressed His-HrsCT1 was detected by western blot with the anti-His antibody. (D) S2 cells were transfected with the indicated constructs followed by immunoprecipitation and western blot to examine the levels of Smo phosphorylation that was recognized by the anti-SmoP antibody. (E) An in vitro kinase assay was performed and then a GST pull-down assay was carried with the bacterially expressed His-HrsCT1 in order to examine whether phosphorylation change the interaction property of Smo. GST-Smo^656−678SD^ bearing phospho-mimetic mutations was also used in this experiment. The same amount of His-HrsCT1 put in the system was detected by a western blot with anti-His. (F) S2 cells were transfected with HA-Hrs and Myc-Smo or Myc-Smo^SD123^ followed by OA treatment. Cell extracts were immunoprecipitated with the anti-Myc antibody and subjected to a western blot with indicated antibodies to detect the Smo-bound Hrs and the levels of Smo expression. The detection of HA with the lysates indicates the expression of Hrs.

To determine the Smo domain responsible for interaction with Hrs, we carried out co-immunoprecipitation experiments and found that Smo cytoplasmic tail (C-tail) but not transmembrane domain interacted with Hrs. To further map Smo domain that interacts with Hrs, we tested various SmoCT truncations generated previously [27]. We found that deletion of amino acids 679–730 dramatically reduced and deletion of amino acids 679–764 completely abolished the interaction with Hrs (Fig. 6A, right panel). We then made series internal deletions in Smo in order to verify the interaction at the full-length background (Fig. 6B, the schematic drawing). HrsNT1 interacts whereas HrsNT2 did not interacts with Smo (Fig. 4A), thus we use HrsNT1 to determine the Hrs UIM domain interaction with Smo. To examine the VHS and FYVE domain interaction with Smo, we used Hrs^ΔU^. After series of co-immunoprecipitation experiments (Fig. 6B, lower panel), we found that HrsNT1 interacted with Smo aa679–753 whereas Hrs^ΔU^ interacts with Smo aa661-678. Consistently, HrsCT1 (the VHS and FYVE domains of Hrs) interacted with Smo aa661-678 (not shown). These data suggest that the UIM and N-terminal domains of Hrs interact with different region of Smo (Fig. 6B, summarized in top panel).

Phosphorylation promotes Smo cell surface accumulation, whereas dephosphorylation leads Smo endocytosis [12], [28]. To explore whether Hrs has a role in regulating Smo phosphorylation, we carried out in vitro kinase assay with bacterially expressed proteins. As shown in Fig. 6C, GST-tagged Smo aa656-678 was efficiently phosphorylated by kinases, which was detected by the anti-SmoP antibody that recognized Smo phosphorylation by PKA and CK1 (Fig. 6C, top panel). The addition of bacterially expressed HrsCT1 blocked Smo phosphorylation, suggesting that HrsCT1 binds Smo and masks Smo phosphorylation (Fig. 6C, top panel). Consistently, the overexpression of HrsCT1 blocked the basal as well as the Hh-induced phosphorylation of Smo in S2 cells (Fig. 6D, top panel). These data suggest that interaction between Smo phosphorylation clusters and Hrs N-terminal domains prevents Smo activation by inhibiting Smo phosphorylation.

We showed that Hh downregulates the interaction between Hrs and Smo (Fig. 3B-C). We thus wondered whether phosphorylation of Smo reduces Smo-Hrs interaction. Using a GST-fusion protein pull-down assay, we found that phosphorylated forms of GST-Smo pulled down a little His-tagged HrsCT1 (His-HrsCT1), whereas the unphosphorylated forms of GST-Smo pulled down much more His-HrsCT1 (Fig. 6E, top panel). In addition, the phospho-mimetic mutation completely abolished GST-Smo interaction with His-HrsCT1 (Fig. 6E, top panel). These data suggest that phosphorylation of Smo counteracts Smo interaction with Hrs. In a similar GST pull-down assay, His-tagged Hrs UIM domain (His-HrsUIM) did not interact with GST-Smo regardless phosphorylation (not shown), suggesting that UIM domain interacts with Smo indirectly. In cultured S2 cells, the interaction between Myc-Smo and HA-Hrs was blocked by the treatment of okadaic acid (OA), a phosphatase inhibitor that elevates Smo phosphorylation (Fig. 6F, top panel). OA treatment blocked Smo ubiquitination (not shown), similarly to that caused by phospho-mimetic mutation in Smo [13], [14]. These data suggest that phosphorylation dissociates Smo from Hrs interaction. Compared to wild-type Smo, Smo^SD123^ that has phospho-mimetic mutations in the residues of three phosphorylation clusters interacted with Hrs weakly and such interaction was completely blocked by the treatment with OA (Fig. 6F, top panel), suggesting that phosphorylation of Smo in cultured cells blocks its interaction with Hrs. Since Smo^SD123^ still has very weak interaction with Hrs and OA treatment completely blocks this weak interaction, it is possible that phosphorylation of the residues outside the three clusters in Smo also reduces Smo-Hrs interaction.

### Tsg101 Regulates Smo Trafficking in the Late Endosomes

**Figure 7:**
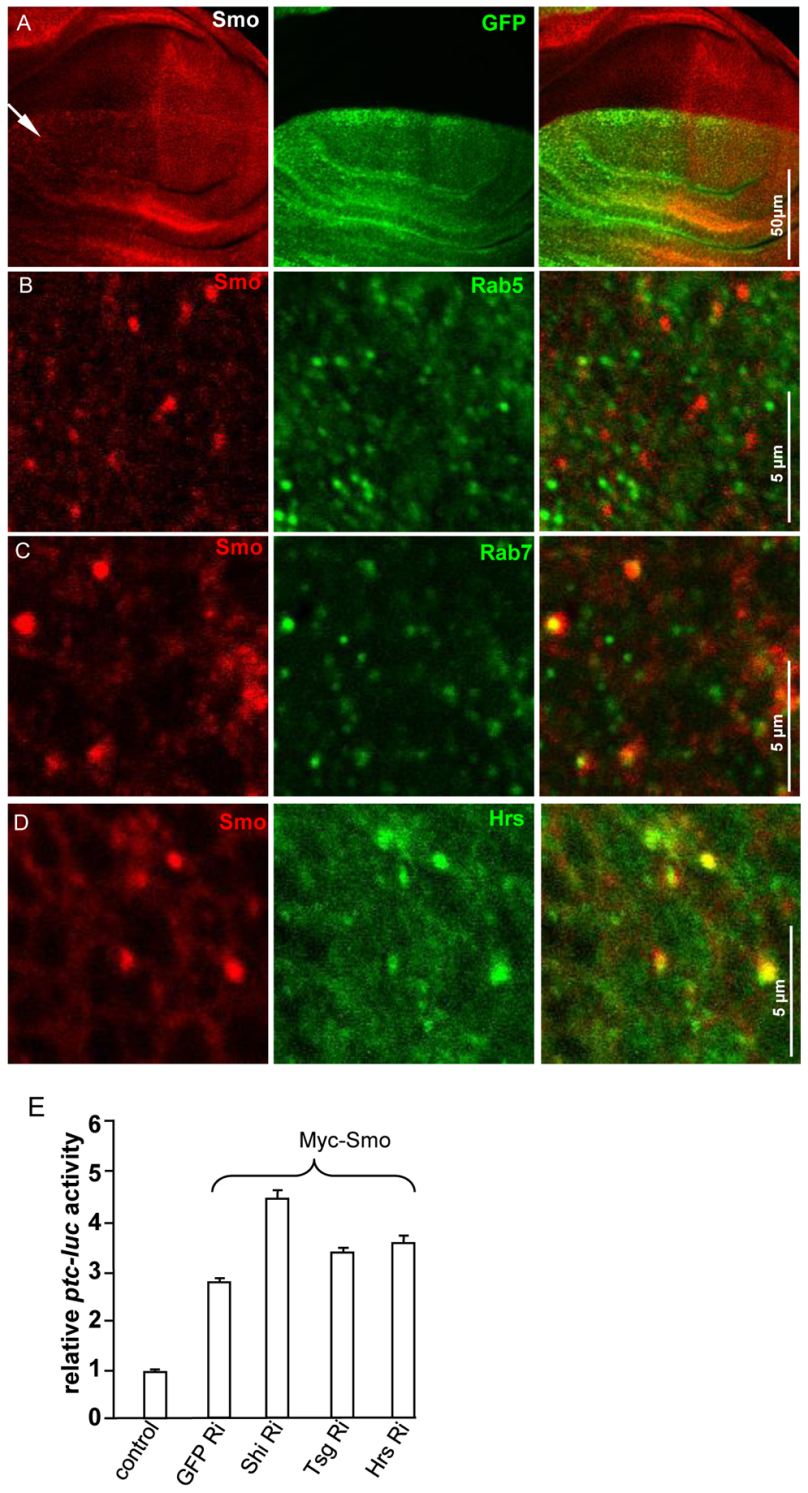
Smo regulation by Tsg101. (A) A wing disc expressing Tsg101RNAi by *ap*-Gal4 was stained for Smo. GFP labels the dorsal compartment cells that express Tsg101RNAi. (B) Large magnification of a wing disc expressing Tsg101RNAi by *ap*-Gal4 and stained for Smo and Rab5. The accumulated Smo puncta do not co-localize with Rab5. (C) Large magnification of a wing disc expressing Tsg101RNAi by *ap*-Gal4 and stained for Smo and Rab7. (D) Large magnification of a wing disc expressing Tsg101RNAi by *ap*-Gal4 and stained for Smo and Hrs. The accumulated Smo puncta by Tsg101RNAi co-localize with Rab7 and Hrs. (E) S2 cells transfected with Myc-Smo followed by RNAi of the indicated endosomal components were subjected to luciferase assay with cotransfection of *tub-Ci* and the *ptc-luc* constructs. GFP RNAi served as control RNAi. RNAi efficiency was confirmed by western blot with anti-GFP or anti-Flag antibody for tagged Shi, Tsg101, and Hrs (not shown).

After endocytosis from the plasma membrane into early endosomes, ubiquitinated receptors are bound to the ESCRT-0 complex that contains Hrs [26], [29]. Hrs selects ubiquitinated cargos and recruits components of other ESCRT complexes including ESCRT-I, II and III. Tsg101, a subunit of ESCRT-I, binds the Hrs-receptor complex and recognizes ubiquitinated cargoes. To determine whether Tsg101 is also involved in Smo trafficking, we turned to the *Drosophila* imaginal disc to examine whether inactivation of Tsg101 affect Smo accumulation. We found that knockdown of Tsg101 by RNAi accumulated Smo in A compartment cells (Fig. 7A) leading to Smo puncta (Fig. 7B). However, Smo puncta did not reside in the early endosomes that were labeled with Rab5 (Fig. 7B). Instead, Smo puncta were co-localized with both the late endosome marker Rab7 (Fig. 7C) and Hrs (Fig. 7D). Consistently, Smo puncta co-localized with Rab7-GFP (not shown). These data suggest that Tsg101 regulates Smo in late endosomes.

To examine whether Tsg101 regulates Smo activity, we expressed Tsg101RNAi with Smo^−PKA12^ in the wing and found that the intervein fusion phenotype was enhanced (Fig. 2I). However, Tsg101RNAi alone driven by *C765*-Gal4 did not induce any obvious phenotype (Fig. 2H), even though Tsg101RNAi driven by *MS1096*-Gal4 produced an extreme wing phenotype similar to that caused by HrsRNAi (Fig. 2B, data not shown). These data suggest that inactivation of Tsg101 enhances the dominant-negative activity of Smo^−PKA12^.

To further examine the function of the endosomal compartments in regulating Smo activity, we carried out a *ptc*-luciferase (*ptc-*luc) reporter assay to monitor the activity of Smo in Hh signling. As shown in Fig. 7E, compared to GFP RNAi, Shi RNAi elevated *ptc-*luc activity (Fig. 7E) because the knockdown of Shi increases the cell surface accumulation of Smo [13]. RNAi of Hrs or Tsg101 also elevated the *ptc-*luc activity even though to a lesser extent (Fig. 7E), suggesting that inactivation of Hrs or Tsg101 may not highly accumulate Smo on the cell surface, and that Hrs and Tsg101 act downstream of Shi in regulating Smo intracellular trafficking.

## Discussion

The regulation of Smo intracellular trafficking has been a critical step in understanding the molecular mechanisms of cytosolic Hh signal transduction [20], [28]. In this study, we have identified and characterized the role of Hrs and Tsg101 in the endosomal sorting of Smo. Similar to some other membrane proteins, Smo shares conserved mechanisms by which the multivesicular body (MVB) controls the sorting of ubiquitinated proteins. Novel mechanisms have also been identified in this study. We show that Hrs prevents Smo phosphorylation by directly binding to the phosphorylation sites, which blocks the cell surface accumulation and prevents the activation of the receptor. In addition, Hrs mediates Smo trafficking in the late endosome rather than in the early endosome.

Ubiquitinated membrane receptors are normally internalized through the endocytic pathway and targeted to MVBs and eventually to lysosome for degradation. The ESCRT machinery comprises four protein complexes (ESCRT-0, I, II, and III) that are required for membrane receptors to be sequentially targeted to the MVBs. Although some studies showed that the ESCRT-0 consisting of Hrs can be used as early endosome marker [29], many studies have shown that Hrs mediates the trafficking of ubiquitinated cargos in the late endosome [30]. We found that the inactivation of Hrs from the ESCRT-0 accumulated Smo similarly to the inactivation of Tsg101 from the ESCRT-I, and the accumulated Smo very well co-localized with the late endosome marker, Rab7, suggesting Hrs regulates Smo in the late endosome.

In this study, we found that the ubiquitination is not required for the interaction between Smo and Hrs. Hrs normally recognizes the ubiquitinated cargos through its UIM domain, but the UIM domain obviously does not specifically recognize the ubiquitinated membrane receptor. It has been shown that Hrs, Tsg101, and other component protein from the ESCRTs undergo ubiquitination [31], [32], raising the possibility that Hrs may recognize ubiquitinated proteins from the endosomal sorting machinery. Alternatively, the UIM domain of Hrs might recognize the ubiquitination pathway proteins that carry ubiquitin required for Smo ubiquitination.

We showed that Hrs promotes the ubiquitination of Smo (Fig. 3). It is possible that Hrs competes with USP8 for binding Smo, as Hrs and USP8 directly interact with the same domain of Smo. However, we found that Hrs blocks Smo phosphorylation (Fig. 6C-D), whereas USP8 does not [13], suggesting that Hrs and USP8 bind Smo in different conformation. In support of this hypothesis, manipulating the levels of USP8 does not change the physical interaction between Smo and Hrs (Fig. 4C). In an immunoprecipitation assay, we did not observe a strong physical interaction between Hrs and USP8 (not shown). Hh likely downregulates Smo ubiquitination by promoting Smo-USP8 interaction [13] and by disassociating Hrs (Fig. 3B-C).

It is also possible that Hrs regulates Smo ubiquitination by facilitating the ubiquitin ligase(s). However, the ubiquitin conjugating enzyme(s) and ubiquitin ligase(s) are unknown although it has been shown that mutating the ubiquitin activating enzyme increases the levels of Smo in wing discs [14]. It is also possible that other domains in Hrs help to position the N-terminal direct interacting domain of Hrs to bind Smo. Smo are both mono-ubiquitinated and poly-ubiquitinated at many lysine residues in Smo C-tail [13], [14], [33]. We were unable to narrow down the specific residues that are regulated by Hrs because we found that Hrs promoted the ubiquitination of SmoK13R that contains 13 lysine residues mutated in Smo C-tail [14].

Not only the ubiquitination machinery but also the degradation pathways for Smo have not been clearly addressed, although a recent study has demonstrated VPS36-mediated trafficking of Smo [33]. It is likely that Smo utilizes both the proteasome- and lysosome-mediated degradation pathways [13], [14], [34]. However, it is most likely that Hrs mediates Smo degradation through the lysosome as abolishing Hrs function by mutation or RNAi accumulates Smo in the late endosomes. It is also unknown how ubiquitination directs Smo to the proteasome for degradation. It might involve different types of ubiquitination chain or even might utilize different ubiquitin ligases. The identification and characterization of the ubiquitination machinery will provide helpful information regarding Smo ubiquitination and degradation. Smo is a major therapeutic target since it plays a central role in the Hh signaling pathway, so understanding the mechanisms by which the intracellular trafficking of Smo is regulated may lead to attractive therapeutic strategies.

## Materials and Methods

### Constructs, Mutants, Transgenes

Myc-Smo, Flag-USP8 and a series of Smo truncations including Myc-SmoNT1, NT2, NT3, NT4, and NT5 have been previously described [13], [35]. Myc-Smo deletions including D661-818, D678-818, D753-818, and D678-753 were generated by two-steps PCR with corresponding amino acids deleted. Based on Myc-SmoK6R and K7R [14], Myc-SmoK8R (KR mutations at aa665, 695, 700, 702, 710, 733, 752, and 753) was constructed by site-directed mutagenesis. Full-length Hrs cDNA was obtained from DGRC (#LD30575), amplified by PCR, and inserted into UAST-2×HA vector to generate HA-Hrs. A series of Hrs truncation including HA-HrsCT0, CT1, CT2, NT0, NT1, and NT2 were generated by PCR and inserted into UAST-2xHA vector. HA-Hrs^ΔU^ with the UIM (aa262-282, corresponding nucleotide 784–846) domain deletion was constructed by two-steps PCR. GST-Smo^656−678^ was generated by fusing Smo aa656-678 (corresponding nucleotide 1966–2034) to the GST backbone. His-HrsCT1 and His-HrsUIM were generated by fusing Hrs aa1-240 (corresponding nucleotide 1–720) and aa242-300 (corresponding nucleotide 724–900) to the pET-30a-His backbone, respectively. Fly mutants used: *hrs^D28^* [36]. Hrs RNAi lines were obtained from either Bloomington (#28026 and #28964) or VDRC (v20933), and line #28964 was used for most of the experiments as all those lines gave rise to similar phenotypes. Tsg101 RNAi line (v23944) was obtained from VDRC and was characterized by previous studies [14]. *MS1096* Gal4, *ap-*Gal4, *C765*-Gal4, and *UAS-GFP-Smo* have been described [24], [35]. HA-Hrs and HA-Hrs^ΔU^ transgenic lines were generated at the VK5 attP locus to ensure the proteins are expressed at the same levels without positional effects [27].

### Cell Culture, Transfection, Immunoprecipitation, Western Blot, and Luciferase Reporter Assay

S2 cells were cultured as previously described [11]. Transfections were carried out using Effectene transfection reagent (Qiagen). Forty-eight hours posttransfection, cells were harvested and treated with lysis buffer (100 mM NaCl, 50 mM Tris.HCl (pH8.0), 1.5 mM EDTA, 10% glycerol, 1% NP-40, and protease inhibitor tablet (Roche)). Cell lysate was obtained by centrifuging at 12000 rpm for 10 mins. 6 x 10^6^ cells were harvested and lysed in 450 µl lysate buffer. 50 µl was saved for direct western blots, out of which 4 µl was used for each load. The remaining 400 µl was used for IP assay, which resulted in 30 µl IP samples. 5 µl IP sample was loaded for each run. For immunoprecipitation, cell lysate was added with beads of protein A ultralink resin (Thermo) after adding the proper primary antibody for 2 hours. Then the samples were resolved by SDS-PAGE and transferred onto PVDF membranes (Millipore) for Western blot. About 16 times more of the immunoprecipitation sample was analyzed compared with the corresponding lysate. Western blot analysis was performed using the indicated antibodies and the enhanced chemiluminescence (ECL) protocol. The use of HhN-conditioned medium has been previously described [11]. Treating S2 cells with OA or dsRNA have been previously described [11], [27]. USP8, Shi, and GFP dsRNAs have been described [13]. Hrs dsRNA was synthesized against coding sequence 298–900 and Tsg101 dsRNA was against coding sequence 1–540. The method of using MG132 (Calbiochem), a proteasome inhibitor, and NH_4_Cl (Sigma-Aldrich), a lysosome inhibitor, to block Smo degradation has been previously described [13]. Antibodies used for Western blot: mouse anti-Myc (9E10, Santa Cruz, 1∶5,000), anti-HA (F7, Santa Cruz, 1∶5,000), anti-Flag (M2, Sigma, 1∶10,000), anti-ubiquitin (P4D1, Santa Cruz, 1∶500), anti-GFP (Millipore, 1∶1,000), rabbit anti-GST (Santa cruz, 1∶10,000), anti-SmoP (1∶50) [11], guinea pig anti-Hrs (1∶500) (gift from Dr. Hugo Bellen). The consistency of western blots was confirmed by 3–5 individual repeats. *ptc-luciferase* reporter assay has been described with S2 cells transfected with *tub*-Ci and 3 repeats were analyzed [11].

### In vitro Kinase Assay and GST Fusion Protein Pull-down

For the in vitro kinase assay, GST-Smo fusion proteins were expressed in bacteria, which is harvested, washed with PBS, and suspended with lysis buffer (PBS supplied with 1% Triton X100 and protease inhibitor) at the ratio of 50 µl buffer/1 ml culture. After sonication and centrifugation, glutathione beads were added to the supernatant aliquots (15 µl beads/500 µl lysate), followed by incubation for one hour and washing with PBS for three times at 4°C. The GST fusion protein was then subjected to a kinase assay with commercial PKA and CK1 (New England Biolabs) according to the supplier’s protocols. Phosphorylation of Smo was detected by western blot with the phospho-SmoP antibody that recognizes the phosphorylated forms of Smo [11]. The assay of GST fusion proteins pull-down with His-tagged proteins has been previously described [27].

### Immunostaining of Wing Imaginal Discs

Wing discs from third instar larvae were dissected in PBS then fixed with 4% formaldehyde in PBS for 20 min. After permeabilization with PBT (PBS supplemented with 1% Triton X100), discs were incubated with the indicated primary antibodies for three hours and the corresponding second antibodies for one hour sequentially, and washed with PBT for three times, 20 min per wash, following incubations. Primary antibodies used in this study were as follows: mouse anti-SmoN (DSHB, 1∶10); rabbit anti-β-Gal (Cappel, 1∶1,500), anti-Rab5 (Abcam, 1∶300), anti-Rab7 (gift from Dr. Akira Nakamura, 1∶3000), and anti-Rab11 (gift from Dr. Donald Ready, 1∶3000); guinea pig anti-Hrs (gift from Dr. Hugo Bellen). Secondary antibodies were from Jackson ImmunoResearch Laboratories Inc., affinity-purified for multiple labeling (1∶500). Samples were mounted on slides in 80% glycerol. Fluorescence signals were acquired with the 20 x objective on an Olympus confocal microscope and images were processed with Olympus Fluoview Ver.1.7c. About 15 imaginal discs were screened and 3–5 disc images were taken for each genotype.
